# Development of an Online Asynchronous Clinical Learning Resource (“Ask the Expert”) in Dental Education to Promote Personalized Learning

**DOI:** 10.3390/healthcare9111420

**Published:** 2021-10-22

**Authors:** Rohit Kunnath Menon, Liang Lin Seow

**Affiliations:** 1 Restorative Dentistry, School of Dentistry, International Medical University, Kuala Lumpur 57000, Malaysia; 2 Division Clinical Dentistry, School of Dentistry, International Medical University, Kuala Lumpur 57000, Malaysia; lianglin_seow@imu.edu.my

**Keywords:** e-learning, online learning, dentistry, dental education

## Abstract

This article describes the development and testing of an online asynchronous clinical learning resource named “Ask the Expert” to enhance clinical learning in dentistry. After the resource development, dental students from years 3 and 4 were randomly allocated to two groups (Group A—“Ask the Expert” and L—“lecturer-led”). All the students attempted a pre-test related to replacement of teeth in the anterior aesthetic zone. Group A (33 students) underwent an online case-based learning session of 60 minutes’ duration without a facilitator, while Group L (27 students) concurrently underwent a case-based learning session of 60 minutes’ duration with a lecturer facilitating the session. An immediate post-test was conducted followed by a retention test after one week. Student feedback was obtained. There was a significant increase in the test scores (maximum score 10) for both groups when comparing the pre-test (Group A—5.61 ± 1.34, Group L—5.22 ± 1.57) and immediate post-test scores (Group A—7.42 ± 1.34, Group L—8.04 ± 1.22; paired *t*-test, *p* < 0.001). However, no significant difference was observed in the test scores when comparing Group A to Group L for both the immediate post-test as well as the retention test (Group A—5.36 ± 1.29, Group L—5.33 ± 1.39 (independent sample *t*-test, *p* > 0.05). To conclude, adequately structured online asynchronous learning resources are comparable in their effectiveness to online synchronous learning in the undergraduate dental curriculum.

## 1. Introduction

Learning from clinical cases or case-based learning provides an opportunity for students to demonstrate application of knowledge, thus augmenting the relevance of their learning [1]. Case-based learning promotes inherent motivation to learn, encourages self-directed learning and enhances clinical decision making abilities by repeated experiences [2,3], leading to a profounder understanding and reflection [4]. However, clinical case discussions are usually conducted between a clinical supervisor or lecturer and a group of students in a clinical setting. Clinical learning from clinical cases may also occur during case-based learning sessions conducted by a lecturer for a cohort. In view of the current pandemic, these sessions are routinely being conducted as online synchronous sessions between a lecturer and a group of students. These discussions are usually isolated bundles of learning between a faculty and a group of students. This approach provides restricted opportunity for feedback from other faculty who are not involved in the primary discussion and also precludes the participation from students who are undergoing clinical learning in other cohorts.

Harden and Hart have explained the benefits of e-learning in removing constraints for learning and expanding possibilities [5]. Computer-assisted learning (CAL) provides flexibility for students and teachers by enabling students to choose the time for learning and freeing the time for teachers to focus on topics needing more close supervision [6]. Enhanced accessibility, diminished costs and effective time management have been cited as significant advantages that e-learning may offer as compared to other modes of learning [7,8]. Educational benefits of e-learning have been previously demonstrated in multiple areas including knowledge acquisition, assessment, development of professionalism and also acquisition of physical skills [9,10,11,12]. The concept of developing a “reusable learning package” [13,14] is advantageous in clinical learning, since it provides collaborative learning (learning across semesters/years and disciplines) available anytime and anywhere.

In addition to the development of new e-learning resource to enhance learning, evaluation and comparison of these resource to conventional/traditional methods of learning is equally important. In dentistry, e-learning has been previously found to be equally [6,15,16,17] or more effective [18,19,20] than traditional methods. However, some studies have significant limitations with respect to the method of assessment employed [18], and none of the aforementioned studies have investigated the impact of clinical case-based learning in dentistry on the knowledge acquisition and retention among dental students by employing methodology with minimal bias, thus ensuring reproducibility.

This study describes the development and evaluation of an asynchronous online clinical learning resource and the subsequent evaluation of its effectiveness by a randomized study. This study aimed to compare the knowledge acquisition and retention amongst dental students who utilized the online asynchronous clinical learning resource to those who underwent a lecturer-led learning session with the same content.

## 2. Materials and Methods

### Development of the Online Asynchronous Learning Resource: “Ask the Expert”

**Figure 1:**
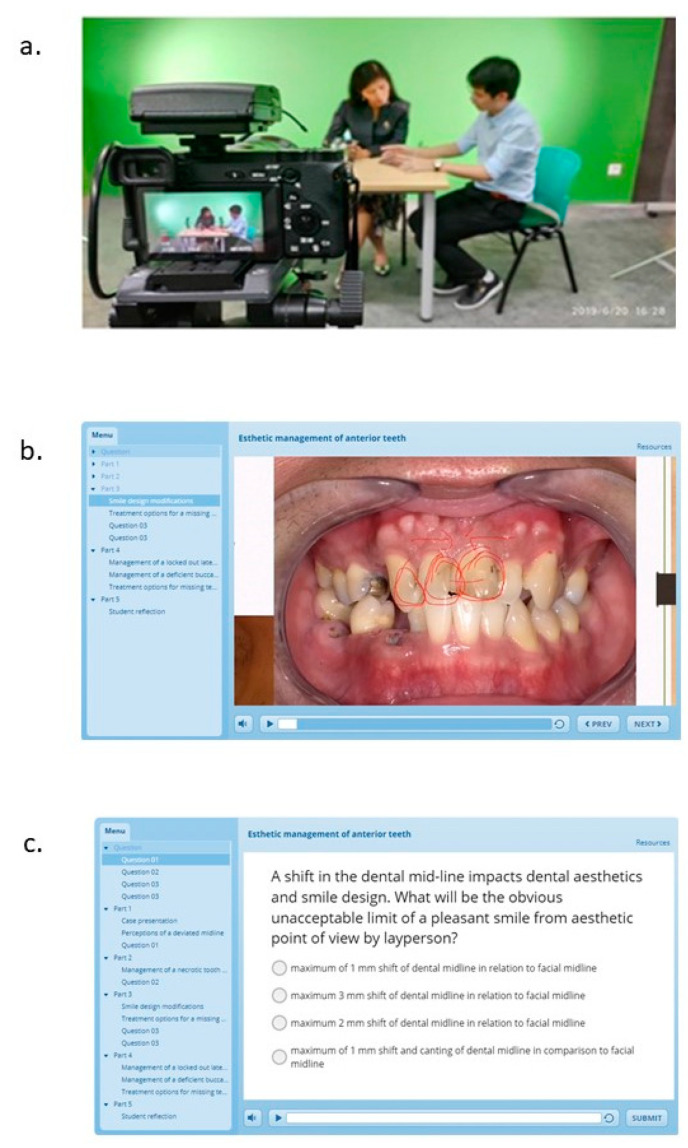
(**a**) Video recording of the case discussion; (**b**) interactive video-based learning resource; (**c**) self-assessment.

We developed an online clinical learning resource named “Ask the Expert”. The portal contains video-recorded clinical case discussions between a clinical supervisor/lecturer and a student. Students are encouraged to contribute clinical cases of interest in a previously provided case template. The case template is a PowerPoint presentation where the areas to enter the relevant patient details and the required photographs and radiographs are indicated. This is provided to ensure a relatively standardized format for case presentations in the learning resource (Supplementary Material Figure S1). The student is required to prepare the case as per the template and store it in a mobile device. Each clinical case is discussed with a clinical supervisor/lecturer using the student’s mobile device (with screen recording) with two additional cameras capturing the discussion (Figure 1a). One camera focuses on the conversation between the student and the lecturer, whereas the other camera focuses on any study models used during the discussion. The discussion between the student and the lecturer is enhanced with the capability to draw on the clinical images and radiographs shown in the student’s mobile device. Upon completion of discussion, the editing team combines the data from the two cameras and the student’s recording on the mobile device to create an interactive video-based learning resource (Figure 1b). Self-assessment components are incorporated into each clinical case in the form of single best answer questions (Figure 1c). At the end of each recorded session, the student is asked to reflect briefly on the discussion with the expert with respect to what they learned. This is included at the end of each video. Further, a forum is created for each case, which is accessible to other students and internal experts for review and discussion. Students and faculty are able to access this anywhere and anytime by scanning a QR code.

At the preliminary phase, the videos are shared with a group of 20 students to acquire preliminary feedback. The videos are re-edited as per student feedback into shorter segments of 1–5 min each. Self-assessment questions are provided in the initial segment before the clinical case discussion commences for each case. When answered incorrectly, students are directed to the section of the video where the correct answer is discussed. The learning resource is a learning bank for clinical cases covering a variety of cases in restorative dentistry. Each case has different learning outcomes, and the self-assessment questions are created from the recorded discussion and then incorporated into the resource.

To evaluate the educational impact of this learning resource, students in years 3 and 4 at the School of Dentistry at the International Medical University, Kuala Lumpur, Malaysia, were invited to participate in the study. The primary outcome of the study was to identify any difference in test scores between the two modes of learning. Ethical approval for the study was obtained from the Joint Committee on Research and Ethics at the International Medical University (Project ID: IMU 480/220). A study information sheet was provided to the students, and the students were given a period of one week to carefully study the information sheet. Students who participated in the creation of the online content were excluded from the study.

The topic covered for the clinical learning session was “Aesthetic restorative dentistry” and, specifically, restoration/replacement of teeth in the anterior aesthetic zone. The learning levels of both the year 3 and year 4 students were assumed to be similar for this topic.

Step 1: Pre-Test

A pre-test comprised of 10 one best answers (OBAs) of one mark each (based on the learning outcomes) were answered by the students who gave written informed consent to participate in the study.

Step 2: Randomization

Subsequently, the students were randomized into two groups, namely Group A (Ask the Expert group) and Group L (Lecturer-led group), by a simple cluster randomization technique. The allocation ratio was 50:50; however, only students who were interested in participating were asked to enroll. The random sequence was computer generated from a random number table. Hence, there was a difference in the number of groups. Consent to participate is an important consideration, especially in education research where students are vulnerable, which was maintained in this context. The learning outcomes and content for the topic were kept standard for both groups to eliminate bias.

Step 3: Intervention

Both the groups underwent the test concurrently during a commonly scheduled time.

Group “Ask the Expert” (A; Online asynchronous learning)—30 students

Students in this group were able to access the online asynchronous learning resource “Ask the Expert” by using a login ID and password, which were provided for each student in the group for 60 min. The resource was uploaded with three clinical cases for the test. The students used the source independently and were not facilitated by a lecturer.

Group “Lecturer” (L; Lecturer-led learning)—27 students

An online synchronous session over Microsoft TEAMs was conducted by a single lecturer with the same clinical cases and content as for Group A for 60 min. The lecturer shared the cases as static PowerPoint slides with the group. After the case was presented, the lecturer instructed the students to answer questions in an OBA format for self-assessment (same as those included in the self-assessment for Group A). This was followed by a discussion between the students and the lecturer regarding the clinical case. The lecturer maintained the discussion similar to the content in Group A, ensuring that the content delivery was standardized. The session was recorded.

Both the online asynchronous session for Group A and the online synchronous session for Group L were conducted concurrently.

Step 4: Immediate post-test

Upon completion of the sessions, an online test was conducted for both groups concurrently, where 10 OBAs were to be answered in 20 min. This was the immediate knowledge acquisition test. The questions used in the immediate knowledge acquisition test were the same as in the pre-test.

Step 5: Retention Test

Both groups were provided with additional reading material including journal articles related to the topic covered. One week after the immediate test, a retention test was conducted for both groups (10 OBAs in 20 min). The questions in the retention test were new questions that included content discussed in the earlier session and information from the shared reading material. However, the newly prepared questions were aligned with the learning outcomes. The primary outcome of the study was to identify any difference in test scores between the two modes of learning.

After the completion of the retention test, all the students were provided access to the asynchronous clinical learning resource “Ask the Expert” and the recorded synchronous sessions to ensure fairness.

Step 6: Student feedback and evaluation of the “Ask the Expert” resource

Student feedback was obtained using a previously validated questionnaire [16]. Various Likert scales were used to test the students’ beliefs about acceptability (Q1), effectiveness (Q2–5) and learning preferences (Q6–7). A section was provided for open comments.

## 3. Results

The average time taken for the development of a clinical case as a learning resource was calculated to be 180 min. The time calculated included the contribution by the student, the lecturer and the personnel involved in editing and uploading the content (Supplementary Material, Figure S2).

The mean and standard deviation of the scores in the pre-test, immediate post-test and the retention test obtained by the students with the pertinent analysis are depicted in Table 1.

The distribution of the scores for both groups are provided in Supplementary Material, Figures S3 and S4.

There was no significant difference in the test scores at baseline (pre-test) between the two groups (Group A—5.61 ± 1.34, Group L—5.22 ± 1.57; independent sample *t*-test, *p* = 0.406). There was a significant increase in the test scores for both groups when comparing the pre-test and immediate post-test scores (Group A—7.42 ± 1.34, Group L—8.04 ± 1.22; paired *t*-test, *p* < 0.001). No significant difference was observed in the test scores when comparing Group, A to Group L for the immediate post-test scores (independent sample *t*-test, *p* = 0.395).

We did not find a significant difference when comparing the pre-test scores to the scores of the retention test (Group A—5.36 ± 1.29, Group L—5.33 ± 1.39; paired *t*-test, *p* > 0.05). No significant difference was observed in the test scores when comparing Group A to Group L for the scores in the retention test (independent sample *t*-test, *p* = 0.788). The distribution of the scores for both groups for the pre-test and immediate post-test are depicted in (Supplementary Material Figures S1 and S2).

The questions used for all the tests are provided as Supplementary Material, Figure S5.

Student feedback was obtained in the domains of acceptability of the learning resource, and its effectiveness and the learning preferences of the students are depicted in Table 2. A total of 52% of the students (30/57) responded to the questionnaire. All the respondents found the method to be acceptable; 93% of the respondents rated the resource as good/very good, and 87% of the respondents indicated that the resource stimulated them to explore the topic further. A total of 60% of the respondents found the method to be time-efficient, 30% were neutral in relation to this question and 10% did not find the resource to be time-efficient.

A total of 63% of the respondents indicated that they would recommend the resource, while the rest remained neutral on this question; 60% of the respondents mentioned that they prefer learning from books, while 37% indicated online resources as the preferred method. A total of 70% of the respondents mentioned lectures as the preferred method, with the remaining indicating private study, e-learning and other methods.

## 4. Discussion

The need for the development of an online asynchronous clinical learning resource emerged from the inability of faculty and students who were not participants in a clinical case discussion to learn from and more importantly contribute to the discussion. A key factor which dictated the demand was feedback from students regarding lack of opportunities to learn from clinical cases being treated by their peers in different cohorts.

Provision of a standard case template was deemed necessary to enable standardization of presentation of cases and minimize preparation time. The students were encouraged to volunteer and share their own cases for discussion. Clinical learning may become more meaningful for dental students when they delve into their own experiences or clinical cases and learn from the content. This approach aligns with the theory of constructivism initially worked on by John Dewey, which proposes that learning is inherently related to action-knowledge, and when students extract learning from their own experiences, it may provide more meaning and significance to the learning [21]. Moreover, learning from one’s own cases and cases treated by peers and faculty in the institution may lend a dimension of authenticity to the learning process, which may be absent in routine learning from the internet or textbooks.

The video-recorded case discussion with the expert marks the next step in the development of resource for clinical case learning. Interaction with the experts contributes to the learning process, where students are exposed to the thinking process of the expert during decision making. This mode of learning aligns with the concept of social constructivism emphasized by Jean Piaget and Lev Vygotsky. Profounder understanding may be achieved by the discussion, increasing the ability of the students to test their own ideas and synthesize and analyses the ideas of others [22,23]. Expert–student dialogue has been previously shown to enhance retention of knowledge and stimulate thinking in undergraduate dental students [24]. With respect to competency assessment in dentistry, expert–student dialogue has been previously shown to result in higher confidence and preparedness, leading to diminished uncertainty and stress. The aforementioned have been reported to contribute to the development of higher-order thinking and a broader clinical experience [25].

Self-assessment in the form of one best answers was incorporated at the commencement of each clinical case, and the same questions re-appeared after the segment of the video in which the answer to the question was discussed by the expert. Self-assessment has been previously established as an integral component of student learning through various studies conducted in dentistry [26,27,28,29,30,31,32]. Self-assessment may enable the students to understand and gauge their thinking and devise strategies to improve in this domain.

Another key element of each clinical case recording was a section on student reflection, where the student reflects on the learning after the completion of the discussion with the expert. After the video recording, the student summarizes and reflects on the discussion with the expert briefly. Reflective learning enables the student to critically review their own experience [33] and connect their current experience with previous learning and build on deeper learning. The incorporation of reflection as a component in the video segment is likely to facilitate deeper learning and critical thinking [34]. Observing a peer performing a reflective discourse (when other students watch the video) gives an opportunity for other students to reflect on and compare their own thought process while critically evaluating the clinical case. Further, the students and faculty may utilize the interactive forum to contribute to a discussion on the clinical case and share their views and experience. This helps to create an avenue for transparency in decision making in the institution and sharing of evidence-based resources in support of the decision or otherwise. Apart from internal faculty, external faculty when visiting as external examiners were also invited to participate in the clinical case discussion. This facilitated collaborative learning with faculty from an external university and hence provided a unique opportunity for the students. 

It takes time, effort and money to generate computer-assisted learning (CAL) tools [35,36]. The development of a completed clinical case video takes 3 h. This includes contribution time from all the contributors, students, faculty and the e-learning department. The reusable learning object thus developed can be used by students and faculty anywhere and at any time and provides unique advantages. Sharing of learning resources and co-operation between universities can lead to economic advantage in the long run. CAL enables standardization of learning material delivery as compared to traditional methods of teaching, which involve different lecturers. Further, improving the interactivity, repeatability and feedback in the CAL program may increase their effectiveness. Real-time feedback and increased interactivity has previously been shown to enhance learning [37,38]. Interactivity incorporated into a CAL program might even be better in holding a student’s attention when compared to traditional methods. Considering the advantages of CAL, new strategies to incorporate these into the curriculum and hence augment/replace conventional teaching should be deliberated.

The randomized study was conducted to evaluate the effectiveness of the current resource in teaching a topic in aesthetic restorative dentistry: “Replacement of teeth in the anterior aesthetic zone”. Two cohorts were invited to participate in the study, and the current learning levels of both the cohorts were assumed to be similar for the specified learning outcome. There may be differences in the knowledge levels of year 3 and 4 students; however, the scope of the learning resource is aligned for all clinical semesters and hence addresses topics with considerable overlap. The scores from the pre-test were not significantly different for both groups, and hence the assumption of baseline comparability was confirmed. Previous studies in dentistry comparing an e-learning intervention to a traditional method of learning have practiced this approach of conducting a pre-test to ensure homogeneity between the groups being compared [15,19,20,39], and out of these three studies used the same questions for the pre-test and immediate post-test as in the current study [15,20,39]. Ensuring the comparability between the groups at baseline is important to ensure homogeneity, particularly since the students belong to two different cohorts. Clinical learning during year 3 and year 4 involve topics which may be of interest and aligned with the learning outcomes for students across the semesters/years. Conventionally, clinical learning may inadvertently be restricted to a particular semester/year due to the allotment of clinical sessions or case-based learning sessions as per the scheduled timetable. This creates a situation where learning may occur in isolated bundles with inaccessibility for the other cohorts, even though the learning may be relevant to them. The creation of an online asynchronous learning resource was hence aimed at creating unbundled learning, which spans across faculty/students in the institution and beyond. There was a significant increase in the test scores at the immediate post-test, which was conducted immediately upon completion of the session for both groups. Hence, we concluded that both the asynchronous learning resource and the synchronous session with a lecturer were equally effective in delivering the learning outcomes for the session. However, no significant difference was found when comparing the scores between the two groups. Previous studies that have evaluated the effectiveness of e-learning and compared it with other forms of learning have yielded mixed results. Overall, e-learning is either equally [6,15,16,17] or more [18,19,20] effective than traditional methods of teaching. However, the use of different methods of assessment for the two groups as undertaken by Eitner et al. is a significant limitation of the study [18]. It is interesting to note that two [18,20] out of three studies in which e-learning had significantly better outcomes than traditional learning had an element of enhanced interactivity in the e-learning tool in the form of assessments and feedback. It is beneficial to assess both short-term knowledge acquisition and long-term retention in the same cohort, as the examinations and real test of what the student has learned is spaced out by time [6,19,39]. The finding from the study conducted by Silveira is also significant, as it indicates that knowledge retention regarding identification of cephalometric landmarks are significantly better after two weeks when compared to conventional learning [19]. Contrary to this finding, we did not find a significant increase in test scores for both groups at the retention test, which was conducted after one week. This may be explained by the fact that we used newly framed questions that were also based on the reading material provided to both groups after the immediate post-test. Nevertheless, the key finding was that there was no significant difference between the two groups when comparing the scores of the retention test, hence suggesting that the online asynchronous learning resource performed at par with the online synchronous learning session. It is interesting to note that the retention test scores were almost the same as the pre-test scores. This can be attributed to the fact that the questions in the retention test were formulated from topics included in the additional learning material shared with both groups. The students may not have adequately covered the learning material provided, leading to the drop in scores. This may reflect the real-life situation in education, where students need to fortify their learning with additional reading; however, they seldom do so. Over-dependence on content from learning tools alone or lecture notes may not be the best way to develop life-long learning skills. This could be considered as a limitation of the study, and in the future, reminders to refer to the additional learning material and feedback based on the exam performance may be used to motivate the learner to refer to the material.

The response rate for the feedback survey was low and may be attributed to survey fatigue for e-learning courses and other feedback requested by the school. The feedback on the e-learning resource was taken after the resource was made available to both groups after the completion of the study to ensure fairness. Hence, intergroup comparisons were not made. The online asynchronous resource was acceptable and received good ratings from the respondents to the feedback survey. Most of the students gave feedback that the resource stimulated the students to explore the topic further. There is previous evidence that structured educational resources may develop desirable habits, linking curiosity and inquisitiveness in the minds of learners, leading to reflection and mindfulness [40]. Interestingly, when queried about learning preferences, most students still prefer to learn from books and through lectures. This may explain the fact that 63% of the respondents indicated that they would recommend the learning resource and the remaining remained neutral in their response. There is previous evidence on the superior effectiveness of lectures over e-learning in dental education [39]. Even though significant effort is being put into the development and validation of digital and e-learning resources, students may use these resources more when employed as augmentation to conventional methods of teaching and learning.

## 5. Conclusions

The online asynchronous clinical learning resource “Ask the Expert” was as effective as online synchronous teaching by a lecturer for clinical case discussions in restorative dentistry. The resource augments other modes of teaching in delivering learning outcomes related to clinical dentistry. This offers a “reusable learning environment” that provides unbundled learning, self-assessment, opportunity for reflection, discussion among peers and opportunity for collaboration with collaborating universities.

## Figures and Tables

**Table 1:** Test scores from the pre-test, immediate post-test and retention test.

Test	Group	Mean	Standard Deviation	*p* Value, *t* test
Pre-Test	A	5.61	1.34	0.406
	L	5.22	1.57	
Immediate Post-Test	A	7.42	1.34	0.395
	L	8.04	1.22	
Retention Test	A	5.36	1.29	0.788
	L	5.33	1.39	

**Table 2:** Student feedback.

Domain	Question	Possible Responses	No. of Respondents/Student Response
Acceptability	Was the method acceptable to you?	Yes	30, 100%
		No	0
Effectiveness	How would you rate this method?	Very Good	7, 23%
		Good	21, 70%
		Neither	2, 7%
		Bad	0
		Very Bad	0
	The method was time-efficient?	Strongly Agree	3, 10%
		Agree	15, 50%
		Neutral	9, 30%
		Disagree	3, 10%
		Strongly Disagree	0
	The method stimulate you to look up the topic further	Strongly Agree	3, 10%
		Agree	23, 77%
		Neutral	4, 13%
		Disagree	0
		Strongly Disagree	0
	Would you recommend this method?	Yes	19, 63%
		No	0
		Maybe	11, 37%
Learning preferences	Which method do you usually use to learn?	Books	18, 60%
		Journals	4, 13%
		E-learning	1, 3.5%
		Internet	6, 20%
		Others	1, 3.5%
	Which method do you prefer the most?	Lecture	21, 70%
		Seminar	0
		E-learning	1, 3%
		Private study	5, 17%
		Other	3, 10%
Student feedback	Open comments and feedback	Open comments	“Good and effective” “Good intervention” “Proper guidance”

## Data Availability

The data presented in this study are available on reasonable request from the corresponding author.

## Supplementary Materials

The following are available online at https://www.mdpi.com/article/10.3390/healthcare9111420/s1, Figure S1: Case template, Figure S2: Time distribution for video resource generation, Figure S3: Distribution of test scores for Group A, Figure S4: Distribution of test scores for Group L, Figure S5: Questions used for pre-, post- and retention test.

## References

[1] Thistlethwaite J.E., Davies D., Ekeocha S., Kidd J.M., MacDougall C., Matthews P., Purkis J., Clay D. (2012). The Effectiveness of Case-Based Learning in Health Professional Education. A BEME Systematic Review: BEME Guide No. 23. Med. Teach..

[2] Schwartz P.L., Egan A.G., Heath C.J. (1994). Students’ Perceptions of Course Outcomes and Learning Styles in Case-Based Courses in a Traditional Medical School. Acad. Med..

[3] Richards P.S., Inglehart M.R. (2006). An Interdisciplinary Approach to Case-Based Teaching: Does It Create Patient-Centered and Culturally Sensitive Providers?. J. Dent. Educ..

[4] Dupuis R.E., Persky A.M. (2008). Use of Case-Based Learning in a Clinical Pharmacokinetics Course. Am. J. Pharm. Educ..

[5] Harden R.M., Hart I.R. (2002). An International Virtual Medical School (IVIMEDS): The Future for Medical Education?. Med. Teach..

[6] Bissell V., McKerlie R.A., Kinane D.F., McHugh S. (2003). Teaching Periodontal Pocket Charting to Dental Students: A Comparison of Computer Assisted Learning and Traditional Tutorials. Br. Dent. J..

[7] Wong G., Greenhalgh T., Pawson R. (2010). Internet-Based Medical Education: A Realist Review of What Works, for Whom and in What Circumstances. BMC Med. Educ..

[8] Ozuah P.O. (2002). Undergraduate Medical Education: Thoughts on Future Challenges. BMC Med. Educ..

[9] Wilson A.S., Goodall J.E., Ambrosini G., Carruthers D.M., Chan H., Ong S.G., Gordon C., Young S.P. (2006). Development of an Interactive Learning Tool for Teaching Rheumatology—A Simulated Clinical Case Studies Program. Rheumatology.

[10] Choules A.P. (2007). The Use of Elearning in Medical Education: A Review of the Current Situation. Postgrad. Med. J..

[11] Bernardo V., Ramos M.P., Plapler H., De Figueiredo L.F.P., Nader H.B., Anção M.S., Von Dietrich C.P., Sigulem D. (2004). Web-Based Learning in Undergraduate Medical Education: Development and Assessment of an Online Course on Experimental Surgery. Int. J. Med. Inform..

[12] Davis M.H., Harden R.M. (2001). E Is for Everything-e-Learning?. Med. Teach..

[13] Greenhalgh T. (2001). Computer Assisted Learning in Undergraduate Medical Education. BMJ.

[14] Lau F., Bates J. (2004). A Review of E-Learning Practices for Undergraduate Medical Education. J. Med. Syst..

[15] Aly M., Elen J., Willems G. (2004). Instructional Multimedia Program versus Standard Lecture: A Comparison of Two Methods for Teaching the Undergraduate Orthodontic Curriculum. Eur. J. Dent. Educ..

[16] Bains M., Reynolds P.A., McDonald F., Sherriff M. (2011). Effectiveness and Acceptability of Face-to-Face, Blended and e-Learning: A Randomised Trial of Orthodontic Undergraduates. Eur. J. Dent. Educ..

[17] Howerton W.B., Enrique P.R.T., Ludlow J.B., Tyndall D.A. (2004). Interactive Computer-Assisted Instruction vs. Lecture Format in Dental Education. J. Dent. Hyg..

[18] Eitner S., Holst S., Wichmann M., Karl M., Nkenke E., Schlegel A. (2008). Comparative Study on Interactive Computer-Aided-Learning and Computer-Aided-Testing in Patient-Based Dental Training in Maxillofacial Surgery. Eur. J. Dent. Educ..

[19] Silveira H.L.D., Gomes M.J., Silveira H.E.D., Dalla-Bona R.R. (2009). Evaluation of the Radiographic Cephalometry Learning Process by a Learning Virtual Object. Am. J. Orthod. Dentofac. Orthop..

[20] Shapiro M.C., Anderson O.R., Lal S. (2014). Assessment of a Novel Module for Training Dental Students in Child Abuse Recognition and Reporting. J. Dent. Educ..

[21] Silva D. (1977). John Dewey: Implications for Schooling. Am. J. Occup. Ther..

[22] Scott H.K., Cogburn M. (2021). Piaget. StatPearls.

[23] Vasileva O., Balyasnikova N. (2019). (Re)Introducing Vygotsky’s Thought: From Historical Overview to Contemporary Psychology. Front. Psychol..

[24] Botelho M.G., Chan A.K.M. (2021). A Microanalysis of Expert-Student Dialogue Videos: Supporting Preparation and Learning for Clinical Competence Assessment. Eur. J. Dent. Educ..

[25] Botelho M., Gao X., Bhuyan S.Y. (2020). Mixed-Methods Analysis of Videoed Expert-Student Dialogue Supporting Clinical Competence Assessments. Eur. J. Dent. Educ..

[26] Wiener R.C., Waters C., Doris J., McNeil D.W. (2018). Comparison of Dental Students’ Self-Evaluation and Faculty Evaluation of Communication Skills During a Standardized Patient Exercise. J. Dent. Educ..

[27] Habib S.R., Sherfudhin H. (2015). Students’ Self-Assessment: A Learning Tool and Its Comparison with the Faculty Assessments. J. Contemp. Dent. Pract..

[28] Emam H.A., Jatana C.A., Wade S., Hamamoto D. (2018). Dental Student Self-Assessment of a Medical History Competency Developed by Oral and Maxillofacial Surgery Faculty. Eur. J. Dent. Educ..

[29] McKenzie C.T., Tilashalski K.R., Peterson D.T., White M.L. (2017). Effectiveness of Standardized Patient Simulations in Teaching Clinical Communication Skills to Dental Students. J. Dent. Educ..

[30] Kim A.H., Chutinan S., Park S.E. (2015). Assessment Skills of Dental Students as Peer Evaluators. J. Dent. Educ..

[31] Quick K.K. (2016). The Role of Self- and Peer Assessment in Dental Students’ Reflective Practice Using Standardized Patient Encounters. J. Dent. Educ..

[32] Bitter K., Rüttermann S., Lippmann M., Hahn P., Giesler M. (2016). Self-Assessment of Competencies in Dental Education in Germany—A Multicentred Survey. Eur. J. Dent. Educ..

[33] Schwoegl E.N., Rodgers M.E., Kumar S.S. (2020). Reflective Journaling by Second-Year Dental Students During a Clinical Rotation. J. Dent. Educ..

[34] Boyd L.D. (2002). Reflections on Clinical Practice by First-Year Dental Students: A Qualitative Study. J. Dent. Educ..

[35] Lowe C.I., Wright J.L., Bearn D.R. (2001). Computer-Aided Learning (CAL): An Effective Way to Teach the Index of Orthodontic Treatment Need (IOTN)?. J. Orthod..

[36] Bahrami M., Deery C., Clarkson J.E., Pitts N.B., Johnston M., Ricketts I., MacLennan G., Nugent Z.J., Tilley C., Bonetti D. (2004). Effectiveness of Strategies to Disseminate and Implement Clinical Guidelines for the Management of Impacted and Unerupted Third Molars in Primary Dental Care, a Cluster Randomised Controlled Trial. Br. Dent. J..

[37] Brezis M., Cohen R. (2004). Interactive Learning with Voting Technology. Med. Educ..

[38] Uhari M., Renko M., Soini H. (2003). Experiences of Using an Interactive Audience Response System in Lectures. BMC Med. Educ..

[39] Peroz I., Beuche A., Peroz N. (2009). Randomized Controlled Trial Comparing Lecture versus Self Studying by an Online Tool. Med. Teach..

[40] Dyche L., Epstein R.M. (2011). Curiosity and Medical Education. Med. Educ..

