# Supplementary material for: Development of an Online Asynchronous Clinical Learning Resource (“Ask the Expert”) in Dental Education to Promote Personalized Learning
Source: Healthcare (Basel). 2021 Oct 22;9(11):1420. doi: 10.3390/healthcare9111420 (PMC8624543; doi:10.3390/healthcare9111420)
Supplement: Supplementary file 1 [file healthcare-09-01420-s001.zip › Figure S1.pdf]

CASE PRESENTATION

Student name:

Cohort:

PATIENT DETAILS

- Initials: KFYY
- Gender: Female
- Age: 67 years old

CHIEF CONCERN

HISTORY OF PRESENTING CONCERN

MEDICAL HISTORY

- Last medical check up:
- Medical condition:
- Medication:
- Allergies:
- Recent hospitalization:

DENTAL HISTORY

FAMILY HISTORY

SOCIAL HISTORY

EXTRAORAL EXAMINATION

- No facial asymmetry
- Smile line
- Temporomandibular joint:
- Lymph nodes:

INTRAORAL EXAMINATION

- Soft tissues: WNL
- BPE:

2/0/2

2/2/2

TOOTH CHARTING

Quadrant 1

18:
17:
16:
15:
14:
13:
12:
11:

21:
22:
23:
24:
25:
26:
27:
28:

Quadrant 2

Quadrant 4

41:
42:
43:
44:
45:
46:
47:
48:

38:
37:
36:
35:
34:
33:
32:
31:

Quadrant 3

INTRAORAL VIEW

FACIAL VIEW

MAXILLARY OCCLUSAL VIEW

MANDIBULAR OCCLUSAL VIEW

BUCCAL VIEW

LEFT VIEW

RIGHT VIEW

FACIAL VIEW (WITH DENTURES)

MAXILLARY OCCLUSAL VIEW (WITH DENTURES)

BUCCAL VIEW (WITH DENTURES)

LEFT VIEW

RIGHT VIEW

CARIES RISK ASSESSMENT

- Total risk: Moderate

E1 : RISK	
High	
Moderate	
Low	(green)

ELEMENT 1 : PATIENT LEVEL CARIES RISK FACTORS	At RISK (1 = YES / 0 = No)
Head and neck radiation	0
Dry mouth (conditions, medications/recreational drugs/self-report)	0
Brushing less than two times a day and /or the use of non-fluoridated tooth paste	0
Snacking on sugary diet more than 3 times/day	0
Symptomatic-driven dental attendance	1
Health access barriers	0

E2:RISK	
High	
Moderate	(yellow)
Low	

ELEMENT 2: INTRAORAL LEVEL RISK FACTORS	At RISK (1 = YES / 0 = No)
Hypo-salivation/Gross indicators of dry mouth	0
PUFA (Exposed pulp, Fistula, Abscess) – Dental sepsis	0
Restorations in the past 2 years	0
Thick plaque: Evidence of sticky biofilm in plaque stagnation areas	1
Appliances, restorations and other causes of increased biofilm retention	0
Exposed root surfaces	0

TOTAL RISK	

TREATMENT PLAN

DIAGNOSIS	TREATMENT PLAN

QUESTIONS

THANK YOU
