# Supplementary material for: Development of an Online Asynchronous Clinical Learning Resource (“Ask the Expert”) in Dental Education to Promote Personalized Learning
Source: Healthcare (Basel). 2021 Oct 22;9(11):1420. doi: 10.3390/healthcare9111420 (PMC8624543; doi:10.3390/healthcare9111420)
Supplement: Supplementary file 1 [file healthcare-09-01420-s001.zip › Figure S5.pdf]

Questions used for pre-test and post-test

1.A shift in the dental mid-line impacts dental aesthetics and smile design. What will be the obvious unacceptable limit of a pleasant smile from aesthetic point of view by layperson?

- maximum of 1 mm shift of dental midline in relation to facial midline
- maximum 2 mm shift of dental midline in relation to facial midline
- maximum 3 mm shift of dental midline in relation to facial midline
- maximum of 1 mm shift and canting of dental midline in comparison to facial midline

2.How would you manage an asymptomatic central incisor in an adult patient with a previous history of trauma showing an apical radiolucency and an open apex:

- Monitor the tooth to evaluate if it develops any symptoms
- Complete root canal treatment and provide suitable coronal restoration.
- Complete root canal treatment coupled with apical treatment with MTA and provide suitable coronal restoration.
- Complete root canal treatment coupled with apical treatment with calcium hydroxide and provide suitable coronal restoration.

3.A 40 year old patient has requested to replace a missing maxillary first premolar. The maxillary second premolar has a disto-occlusal amalgam restoration while the maxillary canine is sound with no periodontal involvement. Considering the opposing arch contains natural teeth and the patient has poorly controlled diabetes. Which one of the treatment options given below would you recommend for this patient? :

- Resin-bonded bridge
- Cantilever bridge
- Implant-supported crown
- Fixed-Fixed bridge

4.A 55 year old patient has requested to replace a missing maxillary first premolar. The maxillary second premolar has a small mesial composite restoration while the maxillary canine is rotated. The patient is wearing a removable partial denture replacing all the mandibular premolars and molars. Considering that the patient has financial constraints, which one of the treatment options given below would you recommend for this patient? :

- Resin-bonded bridge

- Cantilever bridge
- Implant-supported crown
- Fixed-Fixed bridge

5.Cone-beam computed tomography of a missing tooth region shows a horizontal ridge defect of 4 mm. An implant-supported restoration is planned. The most recommended method for managing the ridge defect would be:

- Soft tissue grafting
- Distraction osteogenesis
- Autogenous block bone grafts
- Guided bone regeneration

6.The most important consideration when choosing a tooth as an abutment for a resin-bonded bridge is:

- Sufficient amount of sound enamel on the abutment tooth
- The occlusal contact on the abutment tooth and the pontic
- Angulation of the abutment tooth
- Periodontal health of the abutment tooth

7.The most frequently encountered complication with a resin-bonded bridge is:

- Aesthetic failure
- Fracture of the ceramic veneering material
- Loss of retention
- Recurrent caries underneath the retainer

8.The main reason for the loss of retention for a fixed-fixed (two retainers) design on a resin-bonded bridge is:

- Poor cementation technique
- Differential movement of both the abutments
- Excessive occlusal contact on excursive movements
- Distortion of the bridge framework

9.What is the critical factor in ensuring the success of a resin-bonded bridge in terms of occlusion

- Light contacts on both the retainer and the pontic
- Progressive contact on the retainer followed by the pontic
- Avoid contact on the pontic

- Provide a mutually protective occlusal scheme

10.The minimum occlusal clearance required for a metal retainer for a resin-bonded bridge is:

- 0.5 mm
- 0.7 mm
- 0.9 mm
- 1.2 mm

Questions used for retention test

1.Which of the following statements regarding a resin-bonded bridge (RBB) is NOT TRUE

- a. The most common cause for failure in an RBB is loss of retention.
- b. A higher success rate is seen for RBBS in the anterior region.
- c. Wrap around design is equally recommended for RBBs in the anterior and posterior region.
- d. RBBs more commonly fail due to caries than periodontal diseases.

2.Which of the following statements regarding a resin-bonded bridge IS TRUE

- a. When two abutment teeth have been used, it is likely that the RBB will debond from both simultaneously.
- b. RBBs are more successful in a cantilever design than a fixed-fixed design with abutments on each side.
- c. When two abutments are used, occlusal force leads to the tooth and the
- retainer being driven apart causing failure of the cement lute
- d. Multiple abutments increase the likelihood of success in a patient exhibiting bruxism.

3.What is the most important factor in determining the clinical success outcome of ceramic veneers?

- a. The type of luting cement use
- b. The amount of enamel for the tooth preparation
- c. The ability to isolate the tooth with rubber dam
- d. The type of ceramic use to fabricate the veneers

4.It is recommended that the pontic of a resin-bonded bridge should be excluded from occlusal contact as much as possible. How would you evaluate the space and ensure adequate space for the pontic for treatment planning?

- a. Clinical evaluation with an articulating paper
- b. Radiographic evaluation
- To do a wax occlusal record for patient and evaluate the wax thickness
- d. Evaluating the diagnostic wax up of articulated casts

5.Which of the following is the most important factor in determining the type of definitive restoration for a root treated tooth?

- a. The occlusal load on the tooth
- b. The final outcome of the root treatment
- c. The amount of remaining tooth structure
- d. If the tooth will be used as abutment for a prosthesis

6.In the situation where a restored tooth needs to be used as an abutment for a resin-bonded bridge, what modifications would you make in your preparation design?

- a. Limit the preparation design to sound enamel
- b. Extend the preparation labially to create a tag
- c. Consider incorporating the composite into the retainer design
- d. Include one additional abutment with sound enamel

7.The advantage that MTA has over calcium hydroxide with respect to apexification of immature permanent teeth is:

- a. Higher clinical success rate
- b. Higher radiographic success rate
- c. Better ability to form an apical barrier
- d. Shorter treatment time

8.All the following are advantages of MTA when used as an apical barrier EXCEPT

- a. Reinforcement of the root canal dentin
- b. Predictable apical barrier formation
- c. Antimicrobial activity
- d. Less cytotoxicity

9.Which of the following deviations in facial symmetry are least noticed by laypersons.

- a. Midline deviation
- b. Canting of the midline
- c. Chin deviation
- d. Canting of the incisal plane

10.The second most common complication associated with resin-bonded bridges with metal retainers are:

- a. Debonding
- b. Chipping of veneering material
- c. Aesthetic failure
- d. Fracture of the bridge framework
