# Supplementary figures and images for: Development of an Online Asynchronous Clinical Learning Resource (“Ask the Expert”) in Dental Education to Promote Personalized Learning
Source: Healthcare (Basel). 2021 Oct 22;9(11):1420. doi: 10.3390/healthcare9111420 (PMC8624543; doi:10.3390/healthcare9111420)

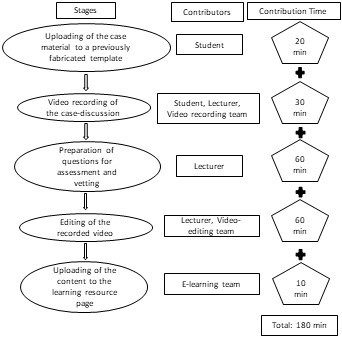

Supplement: Supplementary file 1 [file healthcare-09-01420-s001.zip › Figure S2.jpg]

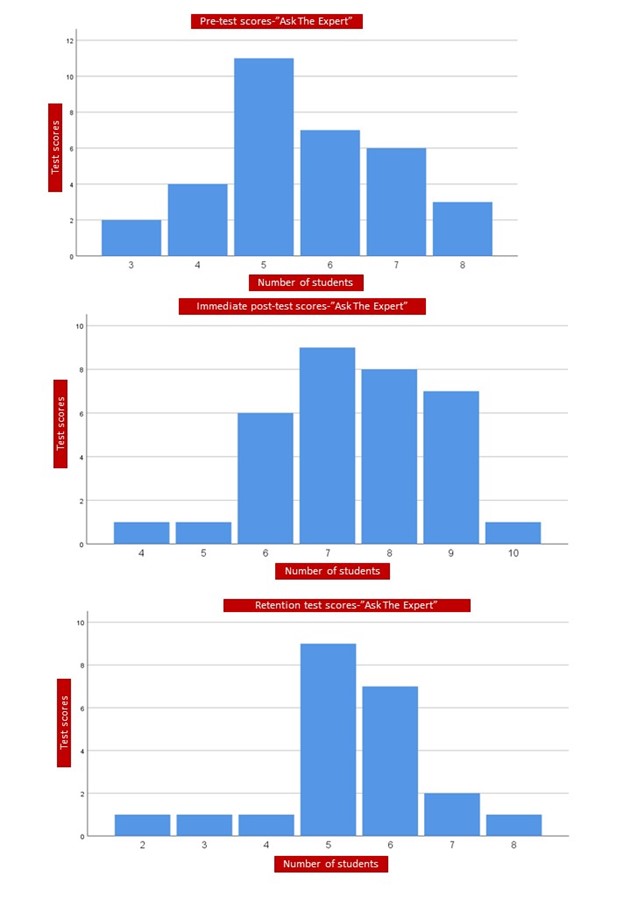

Supplement: Supplementary file 1 [file healthcare-09-01420-s001.zip › Figure S3.jpg]

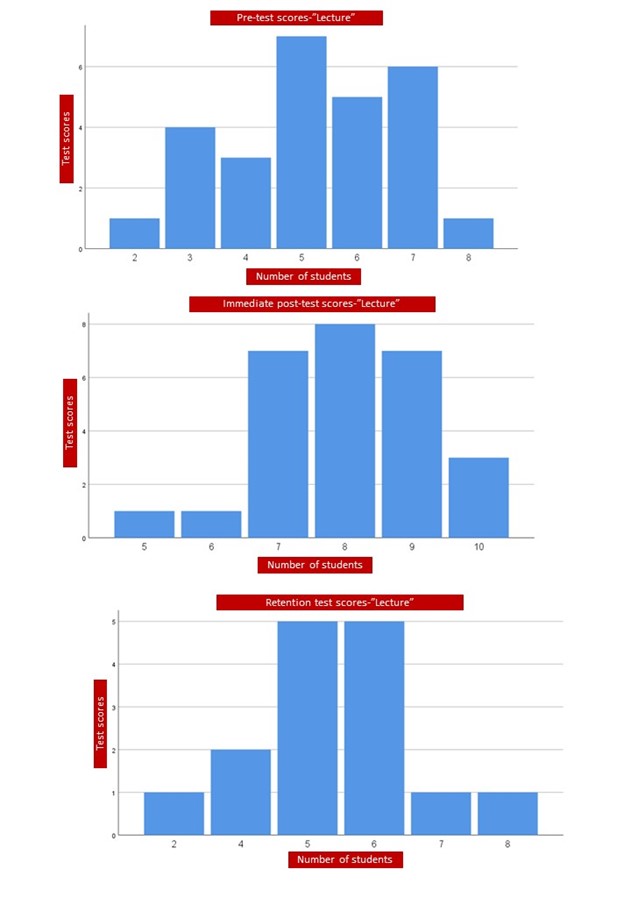

Supplement: Supplementary file 1 [file healthcare-09-01420-s001.zip › Figure S4.jpg]
